# SARS-CoV-2 immunity and vaccine strategies in people with HIV

**DOI:** 10.1093/oxfimm/iqac005

**Published:** 2022-08-17

**Authors:** Claire Mullender, Kelly A S da Costa, Aljawharah Alrubayyi, Sarah L Pett, Dimitra Peppa

**Affiliations:** Centre for Clinical Research in Infection and Sexual Health, Institute for Global Health, University College London Institute for Global Health, London, UK; Division of Infection and Immunity, University College London, London, UK; Division of Infection and Immunity, University College London, London, UK; Nuffield Department of Clinical Medicine, University of Oxford, Oxford, UK; Centre for Clinical Research in Infection and Sexual Health, Institute for Global Health, University College London Institute for Global Health, London, UK; Medical Research Council Clinical Trials Unit, Institute of Clinical Trials and Methodology, London, UK; Division of Infection and Immunity, University College London, London, UK

**Keywords:** COVID-19, SARS-CoV-2, vaccination, HIV, immune responses

## Abstract

Current severe acute respiratory syndrome coronavirus-2 (SARS-CoV-2) vaccines, based on the ancestral Wuhan strain, were developed rapidly to meet the needs of a devastating global pandemic. People living with Human Immunodeficiency Virus (PLWH) have been designated as a priority group for SARS-CoV-2 vaccination in most regions and varying primary courses (two- or three-dose schedule) and additional boosters are recommended depending on current CD4+ T cell count and/or detectable HIV viraemia. From the current published data, licensed vaccines are safe for PLWH, and stimulate robust responses to vaccination in those well controlled on antiretroviral therapy and with high CD4+ T cell counts. Data on vaccine efficacy and immunogenicity remain, however, scarce in PLWH, especially in people with advanced disease. A greater concern is a potentially diminished immune response to the primary course and subsequent boosters, as well as an attenuated magnitude and durability of protective immune responses. A detailed understanding of the breadth and durability of humoral and T cell responses to vaccination, and the boosting effects of natural immunity to SARS-CoV-2, in more diverse populations of PLWH with a spectrum of HIV-related immunosuppression is therefore critical. This article summarizes focused studies of humoral and cellular responses to SARS-CoV-2 infection in PLWH and provides a comprehensive review of the emerging literature on SARS-CoV-2 vaccine responses. Emphasis is placed on the potential effect of HIV-related factors and presence of co-morbidities modulating responses to SARS-CoV-2 vaccination, and the remaining challenges informing the optimal vaccination strategy to elicit enduring responses against existing and emerging variants in PLWH.

## INTRODUCTION

Coronavirus Disease (COVID-19), caused by severe acute respiratory syndrome coronavirus-2 (SARS CoV-2), emerged in the late 2019, and was declared a global pandemic by the World Health Organization (WHO) in March 2020. As of December 2021, >277 million cases and >5 million deaths had been reported, almost certainly a significant under-estimation of the true numbers, and have led to significant pressures and disruption of local, national and international healthcare systems [1]. It has been estimated that People living with Human Immunodeficiency Virus (PLWH) represent ∼1% of total hospitalized cases [2]. However, the actual prevalence of SARS-CoV-2 infection could be higher in low- and middle-income countries where access to diagnosis is limited, and HIV burden is much higher. With nearly 40 million PLWH and 12.6 million people not under suppressive antiretroviral therapy (ART) [3], the dynamics of co-existing SARS-CoV-2 infection require a syndemic understanding of health and disease.

Unlike HIV infection, which in the absence of ART is invariably fatal, the course of COVID-19 disease is highly variable. The majority of cases are either asymptomatic or mildly symptomatic with cough, upper respiratory symptoms, myalgia and headache, but some progress to a potentially fatal condition of acute respiratory distress syndrome, septic shock and multiorgan failure [4–6]. There is an exponential increase in mortality with increasing age [7] and there is a clear correlation between risk of severe disease and comorbidities including hypertension, diabetes, cardiovascular and respiratory disease [8, 9]. PLWH have a higher burden of these disease risk factors than the general population. Furthermore, PLWH are an ageing population, with nearly half of the PLWH in the USA being >50 years of age, which is set to increase [10, 11].

Immunosuppressed patients, including people with haematological malignancies [12], solid organ transplant recipients [13] and those on chronic oral glucocorticoids for rheumatic conditions [14] have also been identified as being at high risk for severe COVID-19 disease. Similarly, PLWH have been included among those deemed vulnerable to worse outcomes from SARS-CoV-2 infection [15]. Large cohort studies from the UK, South Africa, the USA and data reported to the WHO from across the world have identified a higher risk of death and hospitalization from COVID-19 disease in PLWH [16–19]. There is also evidence for a more severe course of COVID-19 disease in people with cellular immune deficiency and a lower CD4+ T cell count/low CD4+ T cell nadir [20–22]. As a result, SARS-CoV-2 vaccination is recommended by national and international HIV societies for PLWH [15, 23, 24]. An informal poll of more than 100 countries from all regions, performed by the WHO, showed that at least 40 countries have an immunization policy that prioritizes vaccinations for PLWH [25]. In general, PLWH and especially those with a CD4+ T cell count <350 cells/µl or ongoing viraemia, are advised to have three doses of vaccine as part of their primary vaccination course [23, 24]. Given that sub-optimal responses to several other vaccines have been reported in PLWH [26], this raises concerns about the potential efficacy of SARS-Cov-2 vaccines in this potentially more vulnerable population. Additional vaccine doses are expected to increase responses in this group, reflected in recommendations by most Western countries, the USA and the UK, advising a first booster (fourth dose) and second booster (fifth dose). These guidelines are regularly updated in line with the evolving pandemic response [27].

Here, we review the complex interplay between HIV and SARS-CoV-2 infection in adults and summarize the knowns and many unknowns of COVID-19 vaccine responses in the setting of HIV infection.

## IMMUNE CORRELATES OF PROTECTION AGAINST SARS-COV-2 INFECTION

Increased understanding of protective immune responses against SARS-CoV-2 infection and disease progression has provided valuable insights for the development and evaluation of vaccines. The humoral immune response to natural infection and vaccination against SARS-CoV-2 has received a lot of attention. Following infection with SARS-CoV-2, a specific humoral response is initiated [28]. Importantly, IgG antibodies which bind to spike protein, particularly the receptor-binding domain (RBD), are more likely to be neutralizing and these have been linked to viral clearance in patients who have recovered from SARS-CoV-2 infection [29]. Non-human primate (NHP) models illustrated protection from reinfection and total protection provided by passive transfer of neutralizing antibodies [30–32]. Indeed, studies in humans have shown that higher anti-spike IgG and neutralizing antibody titres generated following natural infection or vaccination are associated with a lower risk of reinfection [33], symptomatic disease [34] and a positive correlation between clinical severity and SARS-CoV-2 specific antibodies [35]. There is evidence that the timing of IgG anti-spike response may be a critical determinant in survival; Lucas *et al*. showed that deceased patients mounted a robust, specific response, with neutralizing antibodies. However, it was a delay in seroconversion that resulted in poor viral control in these patients [36]. Many studies have also evaluated the impact and timing of serum IgM- and IgA-specific antibodies [37] which have been related to serological diagnosis and prognosis prediction rather than protective effects [38–40]. Although these specific antibody responses can be detected within 2 weeks of initial infection [41, 42], it has been well-documented that humoral immune responses to coronaviruses are variable and short-lived; levels decay post-infection and vaccination after approximately the first month, with a half-life of ∼2 months [43, 44]. The level of neutralizing antibodies required for continuing protection following natural infection or vaccination has not yet been determined; this is further complicated by the emergence of variants of concern (VOC) which have mutations/deletions to the spike protein, particularly in the RBD, which can impact neutralizing sensitivity [45]. This is an important consideration as all of the currently licensed SARS-CoV-2 vaccines are based on the original Wuhan strain [46–48]. Khoury *et al.* developed a predictive model that suggests there is a proportionate response of neutralization titres whereby the lower the initial response to wild-type virus, the greater the impact on vaccine response to other strains [49]. Several studies have shown continued protection against variants following vaccination persisting for ∼6 months with implications for the timing of boosters [50–52]. Although antibody responses wane, class-switched spike-specific and RBD-specific memory B cells can provide a long-lived memory pool that can react rapidly upon reinfection or vaccine boosting [53]. Spike-specific memory B cells have been shown to persist for 6 months to a year following infection [54, 55], with evidence of higher levels of somatic hypermutation, higher binding affinity and neutralizing capacity [56, 57]. Memory B cell responses may even be of higher quality following mild compared to severe SARS-CoV-2 infection, producing more robust responses [58], even when neutralizing antibodies were undetectable. However, recall responses of RBD-specific memory B cells have been shown to decline with age [53, 59].

Increasing evidence supports a protective role versus pathogenic role of T cell immunity against SARS-CoV-2 infection [60]. Although the characterization of virus-specific T cell responses is more technically challenging, an early development of a cytotoxic CD8+ T cell response is associated with significantly milder disease [61–64] and accelerated viral clearance [65–68]. Further indirect evidence of the importance of T cell responses comes from studies of infection in patients with inherited immune defects of antibody responses and from patients receiving B cell depleting therapies in whom robust CD8+ T cell responses contributed to increased survival [69–72]. SARS-CoV-2-specific T cell responses are detected to a range of structural (NP, M, ORF3a, spike) and non-structural (ORF7/8, NSP7, NSP13) proteins following SARS-CoV-2 infection [65, 67, 73–76]. Despite these positive correlations, the exact role of T cell responses, and which epitopes will be the most protective, remain unclear. Following natural infection, the memory phase is dominated by more CD4+/helper T cells with follicular helper T cells (Tfh) correlating with humoral immunity [77, 78]. Experience from SARS-CoV-1 and MERS also suggests that T cell immunity against SARS-CoV-2 may be more enduring [67] and reassuringly largely retained against the highly transmissible Omicron viral variant [79, 80]. Burgeoning evidence also supports a potential role of pre-existing T cell responses in preventing initial infection [81]. Several studies have shown mainly CD4+ T cell responses in up to 50% of samples from blood donors prior to when SARS-CoV-2 appeared in the human population [67, 73, 82–85]. The majority of these T cell responses are to non-spike peptides, including polymerase-specific T cells that were found to expand in abortive SARS-CoV-2 infection [81], but some responses to spike were also reported [73]. It has been proposed that this cross-reactivity is due to previous infection with common cold coronaviruses, which were circulating in the human population prior to 2019 [86], such as human coronavirus HCoV-229E, HCoV-NL63, HCoV-HKU1 and OC43 [87–90]. Kundu *et al.* examined the role of pre-existing cross-reactive T cell responses in protecting SARS-CoV-2 naïve household contacts of patients infected with SARS-CoV-2. In this study, 52 confirmed exposed contacts were investigated, and T cell responses were assessed in both polymerase chain reaction (PCR)-positive (*n* = 26) and PCR-negative (*n* = 26) contacts. The authors found that the initial frequency of human coronaviruses primed cross-reactive T cells, which secrete interleukin-2 (IL-2), are key to protection in contacts who remained PCR-negative [91]. These findings have implications for future vaccine targets, strongly suggesting that the inclusion of non-spike proteins may be essential to increase the breadth of responses, including novel variants in the future.

A limitation of many studies is that analysis of cellular responses has focused on peripheral blood. It is likely that key T cell responses are being underestimated in the lungs and several studies have shown an increase in T cells in bronchoalveolar lavage (BAL) samples from patients with moderate COVID-19 disease compared to patients with severe disease [64, 92, 93]. It is of note that mucosal immune responses are induced during natural infection [94, 95] but there is little evidence to suggest that current vaccines induce mucosal responses [96, 97] without prior SARS-CoV-2 infection [98]. This is an important area that needs further investigation.

## IMMUNOLOGICAL INTERPLAY BETWEEN HIV AND SARS-COV-2

### The immunological landscape of HIV infection and implications for vaccine efficacy

**Figure 1:**
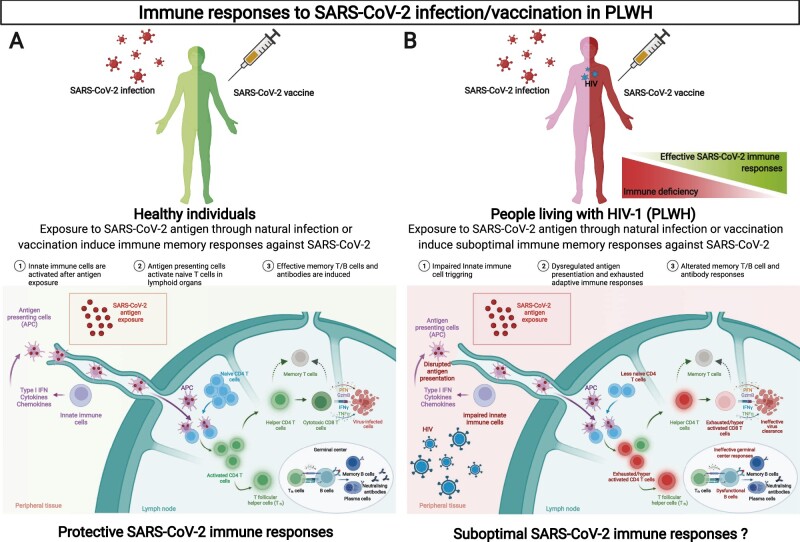
Immune responses to SARS-CoV-2 infection/vaccination in PLWH.

HIV infection induces profound disruption of both the innate and adaptive immune systems (Figure 1). Primary infection induces systemic immune activation and inflammation accompanied by depletion of the T cell compartment, especially in the gut [99, 100]. If left untreated, ongoing viral replication and chronic inflammation leads to the destruction of CD4+ T cells and a persistent expansion of circulating CD8+ T cell numbers. This resulting inversed CD4/CD8 ratio has been associated with frailty and premature ageing of the immune system leading to higher non-AIDS-related morbidity and mortality rates [10, 101–104]. There is an associated reduction in T cell proliferative capacity and cytotoxic potential, which eventually leads to exhaustion [105]. Altered innate immune cell function, such as dysregulation of dendritic cells (DCs), and aberrant responses may also contribute to chronic immune activation and exhaustion [106]. B-cells also develop features of exhaustion relatively early during HIV infection [107]. Abnormal polyclonal activation and poor effector function result in a lack of specific antibody responses, which has been well described [108, 109]. The introduction of ART leads to viral suppression, improved CD4+ T cell counts, and partially restored proportions of B-cell subpopulations [107, 110]. The earlier ART is started, the lower the levels of immune activation and inflammation [111], but despite treatment, chronic activation persists and antigen-specific B and T cell responses, including Tfh cell function, are still impaired [112]. PLWH, despite effective virological suppression, continue to have higher levels of inflammatory mediators, such as IL-6, TNF-α, sCD163, sCD14 and CRP in peripheral blood linked to adverse clinical outcomes [113, 114]. As a result, PLWH are 25 times more likely to suffer from pneumonia and other respiratory diseases, some cancers and infections, such as influenza and tuberculosis, than HIV-negative individuals [115–119]. This raised concerns early in the pandemic that PLWH had a higher risk of infection or a more severe disease course if infected with SARS-CoV-2, despite many PLWH receiving ART, as with other respiratory diseases [120]. Indeed, a more severe disease outcome and increased risk of death have been seen in PLWH, especially when viraemia is not well-controlled or CD4+ T cell count has not been reconstituted sufficiently [20].

These immunological changes and persistent immune dysfunction in PLWH also have implications for vaccination success (Figure 1). PLWH have lower responses to several vaccines including influenza [121, 122], tetanus, diphtheria [123], yellow fever [124] and poliomyelitis [125]. Vaccine responses are better where the CD4+ T cell count has been reconstituted following the commencement of ART [121]. In addition, the total duration of seroprotection is shorter than in otherwise healthy persons for most licensed vaccines [26]. As treatment options have improved, the life expectancy for PLWH have increased. Additional health concerns such as obesity, hypertension and cardiovascular disease, which contribute further to chronic inflammation and reduce vaccine efficacy, have increased [126]. This mirrors the general trajectory of these conditions in the population. Furthermore, ageing is independently associated with senescence of both the innate and adaptive immune systems [127], leading to innate immune cell dysfunction and a reduction in the humoral and cellular responses to several viral and bacterial vaccinations [126]. This age-related loss of immune function, which may be accelerated in PLWH, in addition to changes to the T cell compartment and reduction in the naïve T cell pool, could decrease immune responses to vaccination [112, 121, 122, 128, 129]. Along these lines, the immunogenicity to mRNA [130, 131] and Adenovirus vector [132] SARS-CoV-2 vaccines have been shown to be diminished in healthy subjects over the age of 55 years compared to those under 55 [133, 134]. Elderly individuals also show evidence of reduction in somatic hypermutation of class-switched cells and lower cellular responses following BNT162b2 vaccination [135]. Interestingly, responses were improved following the administration of booster doses [130–132], highlighting that an ageing immune system is a key consideration for the efficacy of currently licensed SARS-CoV-2 vaccines, warranting specific measures to boost responses, especially considering circulating VOCs.

When debating additional factors influencing immune responses to vaccination in PLWH, it is essential to account for the effect of chronic co-infections (e.g. viral Hepatitis B and C). These commonly occur in PLWH and have overtaken other opportunistic infections as the leading cause of death in PLWH [129] and have been linked to a reduction in vaccine efficacy [10, 126, 136, 137]. Co-infection with cytomegalovirus (CMV) is particularly prevalent in PLWH [138]. This contributes to a persistent immune activation state, described herein, through modification of the gut microbiota and microbial translocation, directing responses against itself, and by induction of immune senescence. These factors lead to a decrease in vaccine responses. SARS-CoV-2 vaccination success is also improved when patient CD4+ T cell counts are >350 cells/µl, prior to immunization (Table 1). Similarly, in the case of Hepatitis B vaccination, the CD4/CD8 ratio has proved an accurate predictor of vaccine success [154]. This is not surprising given that a low CD4/CD8 ratio is a marker of immune senescence [155] and therefore may be an important stratification tool to consider as part of vaccination policies for PLWH.

**Table 1:** Summary of SARS-CoV-2 vaccine trial data for PLWH

Vaccine, dose, country and author	Trial design	Participant characteristics	CD4+ T cell count/HIV control (PLWH)	Prior SARS-CoV-2 infection	Immunological readout	Impact for PLWH
ChAdOx1 Two doses UK Frater *et al.* [139]	Phase 2/3	54 PLWH (all male), Median age 42.5 years (IQR 37.5–49.8) 50 HIV negative (24 female, 25 male), Median age 38.5 years (IQR 29.2–45.0)	All PLWH on ART for at least 3 months Median CD4+ T-cell count 694, (IQR 574–860)	Not part of study criteria	IgG spike binding antibody (ELISA) Live virus Neutralization ELISpot T-cell proliferation	Replication deficient adenoviral vector vaccine induces response in PLWH Comparable cellular and humoral responses (magnitude or persistence of response) to HIV-negative participants No correlation between the magnitude of the anti-spike IgG response at Day 56 and CD4+ T cell count (*P* = 0.93) or age (*P* = 0.48)
ChAdOx1 Two doses South Africa Madhi *et al.* [140]	Randomized, double-blind, placebo-controlled, phase 1B/2A trial	103 PLWH 51 Placebo (11 male, 40 female), Median age 41 years (IQR 33–46) 52 Vaccinated (16 male, 36 female), Median age 37 years (IQR 36–46) 58 HIV negative 31 Placebo (19 male, 10 female), Median age 31 years (IQR 26–42) 29 vaccinated (17 male, 12 female), Median age 34 years (range 23–41)	All PLWH on ART for at least 3 months Plasma viral load >100 copies/ml Median CD4+ T cell count 695, (IQR 512–929)	6 HIV-negative participants tested seropositive for SARS-CoV-2 at baseline 8 PLWH tested seropositive for SARS-CoV-2 at baseline	Binding antibody (ELISA) Neutralization	Replication deficient adenoviral vector vaccine induces response in PWLH Participants testing seropositive at baseline had higher levels of spike antibodies and neutralizing titres regardless of HIV status Antibody and neutralization titres increased in all participants following second dose of vaccine
Ad26.CoV2. S Single dose South Africa Khan *et al.* [141]	Participants from the SISONKE South African clinical	26 PLWH (i) Infected unvaccinated *n* = 34 (7 male and 27 female), Median age 41 years (ii) infected vaccinated *n* = 18 All female, Median age 47 years (iii) vaccinated only *n* = 8, (1 male and 7 female) 73 HIV negative between 3 groups	All PLWH receiving ART 10 viraemic SARS-CoV-2 infected and unvaccinated (HIV viral load 1224–30 160 copies/ml), Median CD4+ T cell count 581 1 viraemic SARS-CoV-2 vaccinated (HIV viral load 3219 copies/ml), Median CD4+ T cell count 852 Non-viraemic vaccinated, participants, Median CD4+ T-cell count 735	Actively enrolled unvaccinated and vaccinated participants with prior SARS-COV-2 infection	Neutralization vs. Delta variant only	Participants with well controlled HIV had comparable neutralization of delta variant, regardless of prior SARS-CoV-2 infection Weakest responses seen in unvaccinated PLWH with prior SAR-CoV-2 infection, particularly in those with HIV viraemia
BNT162b2 (mRNA) Two doses USA Woldemeskel *et al.* [142, 143]	Pilot study	12 PLWH (5 male and 7 female), Median age 52 years (IQR 25–59) 17 HIV negative (7 female and 10 male), Median age 41 years (IQR 24–59)	All PLWH receiving ART 3 participants had HIV viral load >20 copies/ml Median CD4+ T cell count 913	No evidence of prior SARS-CoV-2 infection (determined by lack of detectable nucleocapsid antibodies)	CD4 and CD8 ELISpot Anti-spike IgG—ELISA	mRNA vaccine induces antibody responses in PLWH Magnitude and breadth of antibody and T cell responses not significantly different from HIV negative participants, which could be CD4+ T cell count dependent
BNT162b2 (mRNA) Two doses Israel Levy *et al.* [144]	Prospective open study	143 PLWH (131 male and 12 female), Mean age 49.8 years 261 HIV negative (66 male and 195 female), Mean age 55.8 years	All PLWH on ART 95% undetectable HIV viral load Mean CD4+ T cell count at baseline 700 Mean nadir CD4+ T- cell count 345	Not part of study criteria	Binding IgG (RBD)(ELISA) pMN (pseudotype micro-neutralization)	mRNA vaccine induces antibody responses in PLWH Total IgG responses to RBD lower in immunocompromised controls but neutralizing antibodies at similar level to controls Decrease in CD4+ T cell counts observed after each vaccine dose—may impact PLWH with low/unstable CD4+ T cell counts
BNT162b2 (mRNA) Two doses Sweden Bergman *et al.* [145]	Open-label, non-randomized prospective clinical trial	90 PLWH (54 male and 36 female), 79% under 65 years 90 controls (29 male and 36 female), 70% under 65 Additional participants with primary immunodeficiency disorders or secondary immunodeficiency disorders (*n* = 90 per group)	Latest CD4+ T cell count ≤ 300, *n* = 30 Latest CD4+ T cell count >300, *n* = 60	Individuals with prior SARS-CoV-2 infection were excluded	Anti-spike IgG (ELISA)	The primary endpoint was seroconversion rate 2 weeks post second dose 100% of PLWH with CD4+ T cell counts >300 seroconverted following vaccination 96% of PLWH with CD4+ T cell counts <300 PLWH were the only secondary immunodeficiency group that did not have a higher likelihood to seroconvert
BNT162b2 (mRNA)Prime and boost data (two doses total) Germany Jedicke *et al.* [146]	Cohort observational study	PLWH After prime *n* = 88 (75 male and 13 female), Mean age 53.8 years (range 26–86 years) After boost *n* = 52, (39 male and 13 female), Mean age 60.2 years (range 32–85) HCW (controls) *N* = 41 after prime and boost (13 male and 28 female), Mean age 44 years (range 23–61)	Viral load <50 copies/ml, *n* = 84 participants after prime and *n* = 51 participants after boost Viral load of 51–200 copies/ml, *n* = 4 participants after prime and *n* = 1 participant after boost Mean CD4+ T cell count 716 after prime and 577 after boost Mean nadir CD4+ T- cell count 257 after prime and 199 after boost	Not included in study design	Binding IgG and IgA (ELISA) Inhibition by virus surrogate neutralization test (c-pass kit)	All PLWH receiving ART mounted a humoral response regardless of nadir CD4+ T cell count, current CD4+ T cell count, CD4:CD8 ratio after full vaccination. Overall levels of anti-RBD antibodies were variable HIV-negative controls produced significantly higher mean anti-RBD antibody concentrations with less variability
One dose of mRNA vaccines: Moderna (PLWH) or Pfizer (Control group) Canada Nault *et al.* [147]	Cohort observational study 3–4 weeks post-vaccination	106 PLWH (90% male), Mean age 43 years (range 21–65) 20 HIV-negative HCW (healthcare workers), Mean age 47 years (range 21–59)	CD4+ T cell count <250, *n* = 6 CD4+ T cell count 251–500, *n* = 18 CD4+ T cell count >500, *n* = 82 4 participants had detectable HIV viral load	11 participants had seroconverted before vaccination and were excluded from study	Anti-RBD IgG (ELISA)	PLWH with CD4+ T cell counts >250 had comparable antibody responses to control group Lower CD4+ T cell counts resulted in weak responses Study suggests significant association of age single dose vaccine response
mRNA-1273 Two doses Italy Lombardi *et al.* [148]	Prospective single centre cohort	71 PLHW (60 male and 11 female), Mean age 47 years 10 HIV-negative healthy controls (7 male and 3 female), Mean age 58 years	Median CD4+ T cell count 747 Median HIV viral load <50 copies/ml	9 PLWH and 2 healthy controls had prior infection with SARS-CoV-2 (Confirmed by antibodies to nucleocapsid)	Binding IgG (Roche antibody kit) Neutralizing pMN	Vaccination resulted in seroconversion and neutralizing antibody responses in PLWH on ART who were virally suppressed with good CD4+ T cell counts. Neutralizing antibody and anti-S antibody titres were like those displayed by healthy controls, even when stratified according to the CD4+ T cell count PLWH with prior SARS-CoV-2 infection displayed higher anti-S antibody titres (*P* = 0.0007) and neutralizing antibody activity in sera (*P* = 0.0007) than COVID-19-naïve PLWH
Two doses of mRNA vaccines: Moderna (*n* = 9) and Pfizer (*n* = 5) USA Ruddy *et al.* [149]	Prospective observational cohort	14 PLWH only (13 male and 1 female), Median age 62 years (IQR 56–70)	All participants on ART for at least 6 months 13 participants had undetectable viral loads. 1 had detectable viral load (not stated) CD4+ T cell counts: <200, *n* = 2, CD4+ T cell count 200–349, *n* = 1, CD4+ T cell count 350–499, *n* = 3 CD4+ T cell count >500, *n* = 8	Not included in study	Binding IgG antibodies (RBD) (ELISA)	2 doses of mRNA vaccine resulted in high binding antibody titres in PLWH with well-controlled HIV on ART, regardless of CD4+ T cell counts
Heterogenous vs. homologous vaccine schedule Germany Noe *et al.* [21]	Non-interventional, retrospective study	665 PLWH mRNA vaccinated *n* = 590 (492 male, 8 female), Median age 52 years (IQR 43–59) Heterologous schedule *n* = 29 (25 male, 4 female), Median age—56 years (IQR 48–59) AstraZeneca vaccinated *n* = 31 (all male), Median age 31 years (IQR 49.5–63) Janssen vaccinated *n* = 15 (12 male), Median age 46 years (IQR 39.5–59)	Whole study: HIV viral load: 93.5% of participants <50 copies/ml Median CD4+ T cell count 708 Median nadir CD4+ T cell 264	Participants with Prior SARS-COV-2 infection were excluded from the study	Obtained from patient files: Anti-SARS-COV-2 antibody levels (ELISA)	Antibody levels achieved by PLWH following vaccination were comparable to general population mRNA containing vaccination schemes (homo or heterogeneous) had the highest antibody responses Vector-only vaccination scheme had lower median antibody responses Trend towards better responses in female participants Current CD4+ T cell count significantly associated with antibody responses
Heterologous regimens Canada Brumme *et al.* [150]	Non-interventional trial	100 PLWH (88 male, 12 female), Median age 54 years (IQR 40–61) 152 HIV-negative controls (76 male, 76 female), Median age 47 years (IQR 35–70)	Median CD4+ T cell count 710	8 PLWH participants included in study 15 HIV-negative controls included in study	Anti-nucleocapsid and anti-RBD binding antibodies (Roche) ACE displacement assay Neutralization (Live virus)	SARS-CoV-2 vaccination induces binding and neutralizing antibody responses in PLWH on ART with CD4 counts in healthy range Older participants and those with other underlying conditions had weaker responses Vaccination with 1 or 2 doses of mRNA vaccination as part of a 2-dose scheme produces higher peak antibody responses than viral vectored vaccination but waned quicker than 2 doses of ChAdOx1 Increased interval between vaccine doses resulted in high levels of binding antibodies but not neutralizing antibodies
Inactivated whole viral vaccine Prime and boost data (two doses total) China Zou *et al.* [151]	Prospective	46 PLWH (40 male and 6 female), Mean age 38 years 40 HIV-negative controls, Mean age 34 years	Median CD4 count 523 CD4+ T cell count <200, *n* = 2 CD4+ T cell count 200–349, *n* = 8 CD4+ T cell count 350–499, *n* = 11 CD4+ T cell count >500, *n* = 25	Not included in study protocol	Neutralization Binding antibody (IgM and IgG) (ELISA)	Inactivated virus is safe to administer to PLWH PLWH mounted a weaker and delayed humoral response to the inactivated vaccine compared to HIV-negative controls
Inactivated whole viral vaccine Two doses China Lv *et al.* [152]	Interventional Study	24 PLWH (12 male and 12 female), Median age 44 years, 24 HIV negative controls (15 male and 9 female), Median age 37 years.	CD4+ and CD8+ T-cell count levels were enumerated by flow cytometry after vaccination but numbers prior to vaccination not available	Excluded participants with prior history of exposure or infection with SAR-CoV-2	Neutralization (Competitive ELISA) Lymphocyte phenotyping (flow cytometry)	Inactivated whole virus vaccine is safe and capable of inducing neutralizing antibody responses in PLWH The magnitude of neutralizing antibodies was lower compared to HIV-negative participants Lower CD4+ T cell and B cell levels observed following vaccination may explain these difference
Inactivated whole viral vaccine Two doses China Feng *et al.* [153]	Open-label two-arm non-randomized study	42 HIV (29 male and 13 female), Mean age 42.54 years 28 Healthy controls (16 males, 12 females), Mean age 37.79 years	All HIV-positive participants required to have a CD4+ T cell count of >200 at baseline (mean CD4+ T cell count 659) and 4 weeks after vaccination (mean CD4+ T cell count 476.9)	Participants with prior infection with SAR-CoV-2 were excluded	Neutralization (surrogate neutralization assay—Perkin Elmer) RDB binding antibody (ELISA) Lymphocyte phenotyping (Flow cytometry)	Inactivated whole virus vaccine is safe and capable of inducing neutralizing antibody responses in PLWH receiving ART and with a CD4+ T cell count of >200 CD3+, CD4+, CD8+ T Cell counts of PLWH decreased following vaccination but did not lead to clinical adverse events

## IMMUNE RESPONSES TO NATURAL SARS-COV-2 INFECTION IN PLWH

Insights from studies examining the quantity and quality of immune responses in people who have recovered from natural infection with SARS-CoV-2 can help inform the optimization of vaccines. Arguably any underlying differences in cellular compositions (both innate and adaptive immune phenotypes), in addition to uncontrolled viraemia and persistent inflammation in PLWH, could lead to poorly co-ordinated immune responses, affecting the trajectory of COVID-19 disease (Figure 1). Dysregulated immune cell co-ordination has been shown to attenuate protective immune responses in elderly individuals [61], which could be highly pertinent in PLWH with additional co-morbidities. To date, there are limited data on natural immunity following SARS-CoV-2 infection in PLWH from studies which are conducted in high-income countries, and in populations largely controlled on ART.

Given that antibody responses are thought to be an important immune correlate of protection, SARS-CoV-2 IgG levels and neutralizing antibody activity have been compared in PLWH and HIV-negative individuals following natural infection. In a matched case-control observational study involving 955 PLWH and 1062 people without HIV, the SARS-CoV-2 IgG seroprevalence was 3.7% and 7.4%, respectively. Notably, lower anti-RBD IgG and pseudovirus neutralizing antibody titres, with similar avidity, were observed in the HIV-positive group compared with HIV-negative individuals with evidence of past infection [156]. This is in contrast to smaller studies that did not show any difference in IgG concentrations or neutralization potency against SARS-CoV-2 infection in PLWH. Of note, the latter studies included patients who had well-controlled HIV on ART, which may have been a confounding factor [157–159]. Indeed, a correlation between higher CD4+ T cell count and higher neutralization titres in COVID-19 infection has been described in PLWH [141, 160–162]. At present, an in-depth assessment of B-cell-specific memory responses is lacking in the setting of HIV infection.

The role of T cells in SARS-CoV-2/HIV co-infection is still being deciphered. Unpicking the increased risk due to HIV infection rather than the high risk of co-morbidities is challenging. It remains unclear whether HIV-associated immune dysfunction and inflammation are linked to severe COVID-19 disease outcomes [163, 164] or whether paradoxically a low CD4+ T cell count ameliorate disease severity [134]. A recent study by Sharov *et al.* compared the T cell profile and cytokine dynamics of patients with COVID-19 disease with and without HIV infection [161]. Of the 367 patients with HIV, 171 were not on ART due to medication shortages during the pandemic. While a similar T cell response was seen in HIV seronegative and HIV-positive patients receiving ART, patients with uncontrolled HIV infection had an attenuated T cell response. A decline in CD4/CD8 ratio was associated with a poorer disease outcome. As expected, T cells displayed a higher rate of T cell exhaustion in HIV infection, characterized by an increased expression of PD-1 and TIM-3. This was more pronounced in the presence of HIV viraemia, suggesting a synergistic effect of HIV/SARS-CoV-2 co-infection on T cell dysfunction. PLWH in the absence of ART had decreased serum concentrations of IL-2, TNF-α and IFN-γ and higher levels of the immunosuppressive cytokines IL-10 and TGF-β [161]. Findings by Alrubayyi and colleagues showed that PLWH, with well-controlled HIV, in the convalescent phase of predominately mild COVID-19 disease, showed equivalent magnitude of SARS-CoV-2 specific T cell responses compared to HIV-negative individuals, targeting both structural and non-structural proteins [133]. SARS-Cov-2-specific T cell responses were dominated by CD4+ T cells. Remarkably, a positive association was noted between naïve CD4+ T cells, the CD4:CD8 ratio and the magnitude of T cell responses against SARS-CoV-2 in PLWH. These findings suggest that in addition to viraemic HIV infection, inadequate reconstitution of the T cell compartment and fewer pre-existing naïve T cells could hinder the development of memory responses to SARS-CoV-2 infection [157]. Whether dysregulated priming, impairment of Tfh cells and other biological factors not captured in the published studies contribute to poorly co-ordinated humoral and cellular responses remains to be determined.

Remarkably, an increasing number of cases of prolonged COVID-19 infection and/or asymptomatic shedding are being reported in people with advanced immunosuppression [160, 165, 166]. Whilst this underscores the importance of a functional immune response in viral clearance [65] it also has implications for SARS-CoV-2 viral evolution. Prolonged infections provide an opportunity for SARS-CoV-2 to evolve a multitude of mutations, as SARS-CoV-2 mutates at a relatively slow rate compared to other RNA viruses, due the presence of a proofreading mechanism [167]. This was recently demonstrated in an HIV-positive woman with unsuppressed HIV and persistent shedding of SARS-CoV-2 for 210 days, during which time SARS-CoV-2 accumulated 30 mutations, some associated with vaccine escape [166, 168].

Additionally, evidence is emerging that PLWH may be more likely to develop post-acute sequalae or ‘long-covid’ [169, 170] However, an accurate picture of the burden of long-covid in this population remains to be determined, including whether immune cell perturbations described in HIV infection may predispose to long-standing symptoms.

Despite the significant gaps in our knowledge and lack of granular data on immune responses in PLWH with different levels of HIV-related immunosuppression, these findings highlight the need for early access to effective ART and support vaccine prioritization in PLWH. Larger studies are needed, particularly for sub-populations of PWLH (e.g. those with low CD4+ T cell counts) or those with identified high-risk co-morbidities, especially in high HIV burden areas to help inform vaccine recommendations and therapeutics.

## SARS-COV2 VACCINE TRIAL DATA IN PLWH

The Spike glycoprotein has been an excellent target for SARS-CoV-2 vaccines, which have been developed at an impressive speed, as a result of a collective effort by regulatory agencies, pharmaceutical companies and the scientific community [171]. Currently licensed vaccines include mRNA vaccines (mRNA-1273 and BNT162b2) [46, 48], non-replicating adenoviral vectors (ChAdOx1 nCoV-19 and Ad26.COV2.S), viral proteins with an adjuvant (NVX-CoV2373) [172] and inactivated SARS-CoV-2 virus (BIBP-CorV) [153]. Several large Phase 2/3 trials of SARS-CoV-2 vaccines have shown them to be safe and highly effective in the general population. However, after two doses effectiveness reaches 65–90% against infection or mild disease, and 90–100% against severe disease prior to the emergence of VOCs [46–48, 173]. Although individuals with stable treated HIV infection were not excluded in some from the larger Phase 2/3 trials, they made up a small proportion of participants (∼196 for the BNT162b2 mRNA vaccine [48], 176 for the mRNA-1273 mRNA vaccine [46] and 107 PLWH for the ChAdOx1 viral vectored vaccine [47]). Not all the data on PLWH has been presented to date and the small numbers make interpretation on vaccine efficacy difficult. The Ad26.COV2.S trial has included by far the largest number of PLWH (467 people well-controlled with a CD4+ T cell count >300 received a single dose and 498 received a placebo). Two people from the vaccine group and four people from the placebo group developed moderate to severe COVID-19 disease 28 days post-vaccination [174]. It may be that certain vaccine platforms will not be as effective in PLWH or other immunocompromised individuals. Some concerns about the efficacy of NVX-CoV2373 sub-unit vaccine in PLWH have been raised. In one of the pivotal Phase 2a–b trials conducted in South Africa, overall vaccine efficacy dropped from 60.1% to 49.4% when PLWH were included. It is of note that this study was not powered to specifically describe efficacy in the participants with HIV but highlighted the need to specifically assess vaccine efficacy in PLWH [172]. Importantly in this study, 92.7% of sequence cases of SARS-CoV-2 infection accounted for the B.1.351 variant [172]. When assessing vaccine efficacy in PLWH, in addition to numbers included, it is important to consider definitions of efficacy and the epidemiological setting. To date, there are no head-to-head comparisons between vaccines and, as such, whether a certain vaccine platform is more effective in PLWH remains unknown. Future planned studies are planned to address remaining concerns/uncertainties for COVID-19 vaccines in PLWH (NCT04533399; NCT04754698). The main findings of COVID-19 vaccine studies in PLWH are summarized in Table 1.

## VACCINE SAFETY FOR PLWH

Whilst safety concerns surrounding the licensed SAR-CoV-2 vaccines have been publicly voiced and in turn addressed by the scientific community, there have been no additional concerns regarding safety of SARS-CoV-2 vaccinations in PLWH. The most commonly reported side effects include mild local and systemic reactions, and these have been shown to occur equally in PLWH and the general population [175]. There have been some reports of HIV viral blips following mRNA vaccinations. Levy *et al.* highlighted three cases who have low-level viraemia (<100 copies/ml) and a separate case report described a patient who had a viral load of 1760 copies/ml [144, 176] following vaccination. All of these cases had nadir CD4+ T cell counts of <200 cells/ml and/or very high viral loads at diagnosis. However, Levy and colleagues concluded that SARS-CoV-2 vaccination is safe and efficacious in PLWH, with stable CD4+ T cell counts and well-controlled viraemia. Viral blips have been noted with other vaccines, including influenza and hepatitis B, typically 7–14 days following vaccination [177] but these are transient and may be attributed to a reactivation of the latent reservoir. The interplay of SARS-CoV-2 vaccines, the immune system, and latent HIV infection is yet to be thoroughly understood. However, these observations suggest that viral load monitoring post-vaccination may be useful in future studies, particularly for those with low CD4+ T cell counts. It should be highlighted that the benefit of receiving vaccination outweighs the risk.; this key finding is highlighted by the vaccine trials summarized in Table 1.

## SARS-COV-2 VACCINE IMMUNOGENICITY IN PLWH

### mRNA vaccines

Immune responses in PLWH following vaccination with mRNA-based vaccines have been studied more extensively. Two prospective cohort trials [146, 147] and one non-interventional study [150] which compared humoral responses in PLWH and people without HIV found that while the responses to the priming dose of mRNA vaccine were lower in PLWH, following the second dose humoral responses these were comparable to that observed in HIV negative participants. Several small studies have demonstrated excellent seroconversion rates (as measured by detection of spike-RBD specific IgG) with positive responses in 97–98% of PLWH following two vaccines. Notably, these findings were observed in the context of well-controlled HIV [144, 145, 149] with comparable neutralizing antibody titres to HIV-negative people [148]. The requirement for at least two doses of mRNA vaccines was further highlighted by Woldemeskel *et al.* demonstrating equivalent SARS-CoV-2 spike binding antibody titres and cellular responses (assessed by T cell IFN-γ production) irrespective of HIV status. Additionally, there was no significant difference in BNT162b2-elicited SARS-CoV-2 binding antibody levels to the Beta, Alpha and Gamma variants. Despite this, the numbers in this study are small and its findings need to be interpreted with caution [142, 143].

mRNA vaccine immunogenicity is less well-described in PLWH with ongoing immunosuppression and viraemia, who are a particularly vulnerable group that is poorly represented in vaccine trials. In a single case report, lack of seroconversion and no detectable cellular responses were observed following two doses of BNT162b2 in a patient who was vaccinated prior to ART initiation (CD4+ T cell count of 20 cells/µl) [178]. This is consistent with lower seroconversion rates in people with underlying malignancies and transplant recipients [179]. Emerging evidence presented at recent international meetings, indicates that lower CD4+ T cell counts <250 cells/µl, viraemia and/or previous AIDS associate with significantly weaker spike antibody responses, weaker cellular responses and a higher risk of waning neutralizing activity after a median of 5 months in PLWH. This identifies them as more vulnerable to reduced vaccine efficacy [178, 180–183]. PLWH with a CD4+ T cell count <250 cells/µl were found to have a reduced neutralizing ability against the Beta and the Delta variant. No data against Omicron are currently available [183]. As expected, prior SARS-CoV-2 infection predicted higher spike antibodies, as observed for the general population [182]. In an Italian study, a third dose mRNA booster of either BNT162B2 or mRNA-1273 > 28 days following a complete mRNA vaccination course was found to strongly boost humoral responses in PLWH with advanced disease (CD4+ T cell count <200 cells/µl and/or previous AIDS). This was irrespective of the patients’ CD4+ T cell count at the time of boosting and supports the use of an additional vaccine dose in this patient group [184].

## ADENOVIRUS VECTORED VACCINES

The Adenovirus vector-based vaccine ChAdOx1 nCoV-19 has also been shown to induce equivalent humoral responses in PLWH and HIV-negative volunteers. Three published studies compared spike-specific IgG responses and neutralizing antibody profiles of HIV-negative individuals to PLWH with well-controlled HIV and CD4+ T cell counts >350 cells/µl. No significant differences were found based on HIV status [139, 140, 185]. Encouragingly, Madhi *et al.* demonstrated that 50% of PLWH had cross-reactive binding antibodies to the Beta variant and wild-type [140]. High responders retained this neutralization capacity against the Beta variant [139, 140, 185]. Additionally, T cell responses, determined by ELISpot were comparable to the HIV-negative group [140]. Data on the durability of these responses have been recently published showing no significant differences in ChadOx1 nCov19 vaccine-mediated responses, according to HIV status, in 54 PLWH CD4+ T cells >350 cells/µl and 50 HIV-negative age and sex-matched controls. Waning but detectable humoral and T cell immune responses against the wild type and VOCs (Alpha, Beta, Gamma and Delta) were observed 6 months after vaccination [139, 186]. Interestingly in this study, prior exposure to circulating β coronaviruses (HKU1 and OC43) was associated with detectable proliferative SARS-CoV-2 T cell responses at baseline, which were further augmented post-vaccination. This suggests that pre-existing cross-reactive responses could modulate post-vaccine responses in PLWH [186].

Khan *et al*. reported similar neutralization responses in PLWH and HIV-negative individuals who had been vaccinated with a different adenovirus-based vaccine (Ad26.COV2.S) and subsequently became infected with the Delta variant [141]. Whereas PLWH had previously been infected with SARS-CoV-2 and then vaccinated, a 9-fold higher Delta variant neutralization was seen compared to the vaccinated-only group, indicating that vaccination boosted the neutralization response reflecting the same phenomena in the general population [140, 141, 148].

How these data extrapolate to PLWH with lower CD4+ T cell counts and/or ongoing viraemia is not known and additional research is required to address the immunogenicity and durability of adenovirus vectored vaccines in this sub-group of PLWH.

## HETEROGENOUS VACCINATION SCHEDULES AND BREAKTHROUGH INFECTION STUDIES

Optimizing the immunogenicity of vaccines is critical to either stimulate waning immunity or to increase the breadth of immunity. This is either as part of a primary course or against SARS-CoV-2 protein lineage variants, where reduced efficacy has been reported. Data in HIV infection are scarce regarding the optimal vaccination schedule, including the time interval between prime and boost. In the UK a third dose is given as part of the primary immunization course in advanced HIV infection (at least 8 weeks after the last dose) and subsequent booster doses are recommended after the last vaccine dose for all PLWH. In individuals who completed the ChAdOx1 nCoV-19 vaccine schedule, an mRNA booster vaccination is preferentially advised. Thus far, heterogenous vaccination approaches have shown superior immunogenicity outcomes, quantified by both humoral and cellular responses to the wild-type virus and its variants [187]. Both animal studies and emerging evidence in humans, suggest that adenovirus-vectored prime followed by an mRNA boost, at an interval of 6–12 weeks, provides enhanced humoral and cellular responses compared to homologous vaccination [187–192]. In a non-interventional retrospective study, including 665 PLHW in Germany, Noe *et al.* described the anti-SARS-CoV-2 antibody response following standard vaccination (heterologous and homologous) schedules [21]. They found that mRNA vaccination schedules, being female and having a higher CD4+ T cell count were associated with a higher concentration of antibodies in PLWH. There was a markedly lower response in PLWH with a CD4+ T cell count <200 cells/µl, however, as with other studies, the numbers were small. Further studies would be required to confirm if these reduced responses do result in a higher risk of infection and more severe disease. Questions on the optimization of current vaccine schedules and flexibility in using different COVID-19 vaccines were addressed in the Com-CoV2 study in HIV-negative adults aged 50 years and over. These adults were immunized with either: a single dose of ChAdOx1 nCoV-19 or BNT162B2, or heterologous dosing with mRNA-1273 but not NVX-CoV2373. This resulted in increased reactogenicity compared with homologous schedules [193]. Further work is required to address the effects of this mix and match approach prospectively in PLWH with differing levels of immunosuppression and/or natural exposure to SARS-CoV-2 and circulating variants as the epidemic evolves. It is likely that these approaches will add resilience to circulating variants by inducing stimulation of complementary immune pathways, leading to more effective and durable B cell and T cell responses.

To date, few studies have analysed the rates of breakthrough infections in PLWH. Data from Israel has estimated that ∼40% of breakthrough infections occur in immunocompromised individuals [179]. Two large longitudinal cohorts in the USA have estimated a similar number of breakthrough SARS-CoV-2 infections in vaccinated PLWH compared to people without HIV which included 8536 [194] and 31 840 PWH [195]. Both studies found a 33–41% higher risk of breakthrough infection in PLWH, which persisted after regression analysis for covariates such as age, race/ethnicity and sex at birth. Conversely, booster recipients had a reduced risk of infection compared to those who were not boosted, as well as a reduced risk of severe COVID-19 disease outcomes. This indicates that boosters are important tools of protection for PLWH. Interestingly, in contrast to vaccination studies described herein, Coburn and colleagues did not find any correlation between CD4+ T cell count and/or HIV viraemia to be associated with breakthrough risk [195]. However, it should be noted that data on breakthrough infections is limited by diagnostic testing practices and access to healthcare. As with many of the studies included in this review, the duration of ART and the level of suppression required are not consistent between studies and therefore, it is more difficult to untangle the specific effects of these variables and how they may impact vaccine responses.

As for the general population, it is expected that additional vaccine doses will offer some degree of protection against omicron and severe disease requiring hospitalization. Early data from Israel in people aged 60 or older showed that a fourth dose mRNA vaccine against omicron reduces the risk of infection and disease severity. At present HIV-specific data following a fourth (and/or additional vaccine doses) are lacking [196].

## LIMITATIONS OF SARS-COV-2 VACCINES STUDIES IN PLWH

There is currently a lack of standardized assays for determining vaccine efficacy and correlation of protection for humoral or cellular immune responses. The gold standard for vaccine efficacy is neutralizing antibody responses but there are a number of different assays utilized in studies. These include live-virus neutralization [197], pseudotype virus neutralization [197, 198] and surrogate neutralization assays [199]. A consensus on the ideal neutralization assay has not yet been reached as pseudovirus-based assays are not routinely utilized in clinical care. Live-virus neutralization assays are labour intensive and can only be performed by specialist high-containment laboratories with highly trained staff [200]. To address this, several groups have attempted to produce standards, which could be used for comparison of data between labs [35, 201]. This is critical to fully comprehend vaccine responses in PLWH as aggregation of data collected from diverse neutralization, RBD and ELISA assays, and clinical trial designs are required to make statistically significant conclusions. Moreover, the selection of appropriate assays is complicated by the potential for false positives due to interference with anti-retrovirals (e.g. reverse transcriptase and integrase inhibitors), especially in cell-based assays and lentiviral-vector pseudotype virus assays [202]. Additionally, the inclusion of some patients with prior SARS-CoV-2 infection makes interpretation of vaccine response more complex, especially as studies in HIV-negative people have shown that previous infection with SARS-CoV-2 enhances T cell and antibody responses post-vaccination [131, 203, 204].

There are very few studies that focus on the cellular response to SARS-CoV-2 vaccination in PLWH, which may be in part due to technical difficulties in carrying out cellular-based assays. The assumption that the degree of humoral response is paralleled by the cellular immune response may not hold true for PLHW given the distinct T cell dysregulation that occurs. This might be particularly relevant for PLWH with depleted CD4+ T cells, who appear to be at higher risk of severe COVID-19, and reduced responsiveness to vaccine. As with neutralization data, the numbers of PLHW included in published studies are small. Hence, they are unable to adequately adjust for many confounding variables that may affect vaccine responses. In addition, data for SARS-CoV-2 vaccine response for PLWH over the age of 55 are scarce and the combined effect of ageing, chronic illness and HIV infection on vaccines responses is yet to be fully understood, and may in part, account for the findings of higher risk breakthrough infections described in PLWH.

## CONCLUDING REMARKS AND REMAINING CHALLENGES

PLWH have been dealing with a great deal of uncertainty throughout the pandemic, particularly as evidence regarding risk of disease severity has been conflicting, and data on vaccine efficacy remain limited. Studies of seroconversion rates, and neutralization titres post-SARS-CoV-2 vaccination in PLWH, are reassuring for those who have stable HIV on ART and preserved immune function. These findings further highlight the critical role of CD4+ T cells as facilitators of effective humoral responses and offer insights into the complementary role of T cell-specific responses in mediating protection, which may be hindered in people with incomplete immune reconstitution and/or a diminished repertoire of naïve T cells. However, as there is not a consensus on what constitutes protective immunity, it is hard to define protective efficacy in immunocompromised individuals. In particular further work is required to disentangle the importance of T cell immunity in vaccine-mediated protection against SARS-CoV-2 and circulating variants. What is becoming apparent is that PLWH should follow current recommended vaccination schedules and boosters as they become available. This is given that SARS-CoV-2 vaccination is safe and efficacious; overall vaccine effectiveness was 65% (95% CI 56–72%, *P* < 0.001) among vaccinated compared to unvaccinated PLWH [175]. However, these data need to be continuously evaluated in the context of the evolving pandemic, prevalence of circulating variants, different vaccination schedules and number of doses.

Male adults living in Europe, the United States, and South Africa are the most represented participants to date, which poorly reflects the global prevalence of PLWH. Although the primary aim is to start PLWH on ART immediately, this is not always possible in resource-limited settings. The pandemic has further highlighted disparities in access to ART and global disparities in vaccine coverage, which may leave PLWH potentially vulnerable [161, 205]. There is evidence of worse COVID-19 disease outcomes in patients with coinfections, such as Mycobacterium tuberculosis (TB) [16, 17]. The intersecting SARS-CoV-2, HIV, and TB epidemics pose additional concerns, particularly as T cell immunity and SARS-CoV-2–specific CD4+ T cells are reduced and display lower polyfunctional capacity in the setting of co-infection [162].

A potential confounding factor in the evaluation of vaccine efficacy in PLWH is the use of ART as some, i.e. lopinavir–ritonavir, have anti-coronavirus activity *in vitro* [206]. Although the role of ART in preventing complications of COVID-19 has been postulated, it is unlikely that the plasma concentrations of ART are enough to inhibit SARS-CoV-2 replication [207]. Lopinavir–ritonavir has not been shown to reduce inpatient mortality or hospitalization length in patients with COVID-19 and is not currently a recommended therapy [208].

Importantly, it is also becoming increasingly apparent that PLWH represent a diverse population in terms of their immune phenotype and levels of immunosuppression. Specific subgroups could therefore benefit from distinct immunization strategies, such as an adapted vaccine schedule and additional doses to increase protection against severe disease. For instance, altered dose regimens, repeat vaccine series or use of adjuvants may be needed as an additional strategy to improve immunological responses in PLWH with evidence of immunodeficiency or additional co-morbidities, as shown for other vaccines [209, 210]. Assessment of total CD4+ T cell, CD4:CD8 ratios and levels of viraemia should be considered in determining vaccine scheduling and efficacy, with the caveat that it will not capture the full immune profile. Although correlates of protection are currently unknown, spike-antibody ELISA assays are accessible assays and have been shown to correlate with neutralizing antibody responses [29] with the caveat that these responses are reduced against circulating VOCs [211, 212]. Post-vaccination testing for spike antibody could be considered, however, to identify subpopulations of immunocompromised people who may not mount an immune response and therefore require additional protection. Future research should aim to assess the magnitude and the durability of SARS-CoV-2 vaccine-induced antibody and T cell responses in PLWH with particular focus on those with uncontrolled viral infection and/or who have low CD4+ T cell counts to inform the best strategy for boosting. Greater attention needs to be paid to the combined effect of ageing, co-morbidities, and HIV infection as part of the research agenda. Finally, a consensus of assays used for assessment of vaccine responses and a threshold of protection for humoral and cellular responses would greatly benefit assessment of required responses in PLWH.

## Data Availability

All data are contained within the manuscript.
